# Reply to Nyman, U.; Aspelin, P. Regarding Iodixanol for Pediatric CTA. Comment on “Pop, M. Cardiothoracic CTA in Infants Referred for Aortic Arch Evaluation—Retrospective Comparison of Iomeprol 350, Ioversol 350, Iopromide 370 and Iodixanol 320. *Children* 2021, *8*, 949”

**DOI:** 10.3390/children9050709

**Published:** 2022-05-12

**Authors:** Marian Pop

**Affiliations:** 1 ME1 Department, “George Emil Palade” University of Medicine Pharmacy Sciences and Technology of Tirgu Mures, 540142 Tirgu Mures, Romania; marian.pop@umfst.ro; Tel.: +40-749-260-920; 2 Radiology and Medical Imaging Department, Tirgu Mures Emergency Institute for Cardiovascular Diseases and Heart Transplant, 540136 Tirgu Mures, Romania

We greatly appreciate the letter by Nyman et al. [1] to our manuscript titled “Cardiothoracic CTA in Infants Referred for Aortic Arch Evaluation—Retrospective Comparison of Iomeprol 350, Ioversol 350, Iopromide 370 and Iodixanol 320” [2] that describes our experience in using four different contrast agents in pediatric CT angiography for infants referred for aortic arch evaluation.

We agree that obtaining proper contrast enhancements in computed tomography the iodine delivery rate (IDR) is of particular importance, as demonstrated by the detailed studies mentioned [3,4].

Unfortunately, the retrospective nature of our study allowed us to compare images acquired using the Technical Recommendations of the Society for Cardiovascular CT [5] where only flow (mL/s) and volume recommendations were available. Moreover, we would like to note that there was no statistically significant difference between IDRs for different contrast agents.

Per methodology, data were tested for normality using the D’Agostino–Pearson test, with a one-way analysis of variance (ANOVA) or Kruskal–Wallis test used to verify differences between groups. Therefore, in the tables, either mean or median were being displayed. We acknowledge the fact that differences in cardiac output and presence of significant comorbidities are of importance, but unfortunately such data fall outside of the purpose of the study and we can only regret that we have no such data to increase the strength of our results.

We agree with the qualities that allowed iodixanol to be used with great success in patients with impaired kidney function and that it is used with excellent results in the general pediatric CT examinations, and that, as pointed out [1], osmolality is of particular importance in neonates and small children.

Numan et al. [1] correctly pointed out that data regarding the injection site and catheter being used are not available but, historically, a 24- or 26-gauge canula placed in the upper extremities can be used. We can confirm, however, that all examinations were performed using a room-temperature contrast agent; therefore, we postulated that the differences in enhancement might be due to differences in viscosity. This is recognized also by the American College of Radiology guidelines [6] stating that “If a rapid injection rate is desired through a small angiocatheter and if contrast medium viscosity is high, …, the desired injection flow rate may not be achieved”. We would like to thank our colleagues for the suggestion of using a low-viscosity Iodixanol 270, and we are currently working towards its local availability.

We hope that we have cleared any misunderstandings regarding the methodological side of the analysis. CT angiography in infants remains a challenging procedure, especially in patients with congenital cardiovascular diseases, with proper enhancement of the vessels representing a multivariable function.

We agree that we had available a limited sample and we are accepting that we do not know exactly why using similar acquisition and injection protocols with Iodixanol 320 provided up to 40% less enhancement of the large vessels when compared to the other contrast mediums we had available; however, in our opinion, more data and more comparative studies are required to properly establish the justification of its usage in infant CT angiography.

## Data Availability

Not applicable.
